# Comprehensive Study of Honey with Protected Denomination of Origin and Contribution to the Enhancement of Legal Specifications

**DOI:** 10.3390/molecules17078561

**Published:** 2012-07-17

**Authors:** Antonio Iglesias, Xesus Feás, Sandra Rodrigues, Julio A. Seijas, M. Pilar Vázquez-Tato, Luís G. Dias, Leticia M. Estevinho

**Affiliations:** 1 Department of Anatomy and Animal Production, Faculty of Veterinary Science, University of Santiago de Compostela, Lugo, Galicia E-27002, Spain; Email: antonio.iglesias@usc.es; 2 Department of Organic Chemistry, Faculty of Veterinary Science, University of Santiago de Compostela, Lugo E-27002, Spain; Email: xesus.feas@usc.es (X.F.); julioa.seijas@usc.es (J.A.S.); pilar.vazquez.tato@usc.es (M.P.V.-T.); 3 CIMO-Mountain Research Centre, Agricultural College of Bragança, Polytechnic Institute of Bragança, Campus Santa Apolónia, Bragança E-5301-855, Portugal; Email: srodrigues@ipb.pt (S.R.); ldias@ipb.pt (L.G.D.)

**Keywords:** honey, microbiological, palynological, physicochemical, Protected Denomination of Origin

## Abstract

In this study the characterization of a total of 60 honey samples with Protected Denomination of Origin (PDO) collected over three harvests (2009–2011, inclusive), from the Northeast of Portugal was carried out based on the presence of pollen, physicochemical and microbiological characteristics. All samples were found to meet the European Legislation, but some didn’t meet the requirements of the PDO specifications. Concerning the floral origin of honey, our results showed the prevalence of rosemary (*Lavandula pedunculata*) pollen. The microbiological quality of all the analyzed samples was satisfactory, since fecal coliforms, sulfite-reducing clostridia and *Salmonella* were absent, and molds and yeasts were detected in low counts. Significant differences between the results were studied using one-way analysis of variance (ANOVA), followed by Tukey’s HSD test. The samples were submitted to discriminant function analysis, in order to determine which variables differentiate between two or more naturally occurring groups (Forward Stepwise Analysis). The variables selected were in this order: diastase activity, pH, reducing sugars, free acidity and HMF. The pollen spectrum has perfect discriminatory power. This is the first study in which a honey with PDO was tested, in order to assess its compliance with the PDO book of specifications.

## 1. Introduction

According to the European Union Legislation [1], the *Codex Alimentarius* [2] and the Portuguese Law Decree 214/2003 honey is the natural sweet substance produced by *Apis mellifera* bees from the nectar of plants, secretions of living parts of plants, or excretions of plant-sucking insects on the living parts of plants, which the bees collect, transform by combining with specific substances of their own, deposit, dehydrate, store and leave in the honeycomb to ripen and mature. More than 200 substances have been found in honey, the most abundant being carbohydrates (fructose, glucose, maltose, sucrose) [3]. However, minerals, proteins, vitamins, organic acids, flavonoids, phenolic acids, enzymes and other phytochemical substances are also important components of this natural product [4]. It is important to mention that honey’s composition depends on the floral origin, the climate, environmental and seasonal conditions, as well as on its handling and processing [5]. 

It has been used in ethno-medicine since the early humans, and in more recent times it is used in the treatment of burns, gastrointestinal disorders, asthma, infected and chronic wounds. It plays an important role in the human diet, and is also used in pharmaceutical and cosmetic industries [6]. As an easily assimilable food, honey makes a valuable nutritive product for children, athletes and convalescents [4,7]. 

In Portuguese agriculture, honey is a very important product. In fact, there are more than 26,000 beekeepers that produce about 11,000 tons per year of honey [8]. In agreement with the European Regulation [9] in Portugal there are nine Protected Denominations of Origin (PDO) for honey, which demonstrates that besides the investment on the formation of beekeepers, there is a growing interest on the assurance of the quality, with consequences not only in the economic dynamics of the rural areas in which the beekeepers operate, but also in honeys’ commercialization itself. Indeed, the honeys bearing the PDO logo have acquired a high added value, both within national and international level.

In this context, it is mandatory to verify its compliance with the quality specifications of the European Union. Honey’s quality is related to its sensorial, physical, chemical and microbiological characteristics. The physicochemical properties depend on the nectar and the floral source, colour, flavours, moisture, amount of proteins and sugars [10,11]. These quality criteria are well specified by the EC Directive 2001/110 [1], being moisture content, electrical conductivity, ash content, reducing sugars, free acidity, diastase activity and hydroxymethylfurfural (HMF) content the major parameters of interest. However, EU legislation lacks specifications concerning microbial contamination and hygiene of the product. Indeed, many studies have been carried on the physicochemical parameters of honey all over the World [12,13,14,15,16,17,18], but few data about its microbiological safety is available, being the scarce studies essentially devoted to the detection of *Clostridium botulinum*, since honey is the only known dietary reservoir of spores of this Gram-positive bacteria [14,19]. In this context, and considering that honey has several sources of microbial contamination, among them, the pollen, the digestive tract of the *Apis mellifera* bees, dust, air, soil and the processing of the product, it is very important to assess the commercial quality and microbiological safety of honey (counts of moulds and yeasts, spores of *Bacilus* spp. and *Clostridium* spp.).

Thus, the present study comprised several objectives. First of all, it aimed to characterize, in respect to floral origin, physicochemical parameters, microbiological safety and commercial quality, honey with PDO from the “Terra Quente” and from three different harvests: June of 2009, 2010 and 2011. The second goal was to test whether the product met the PDO products’ specifications. Last but not least, contribute to the introduction in the “book of specifications” of some parameters/analyses that are currently neglected: pH and microbiological indicators of safety and quality.

## 2. Results and Discussion

### 2.1. Pollen Analyses

**Figure 1:**
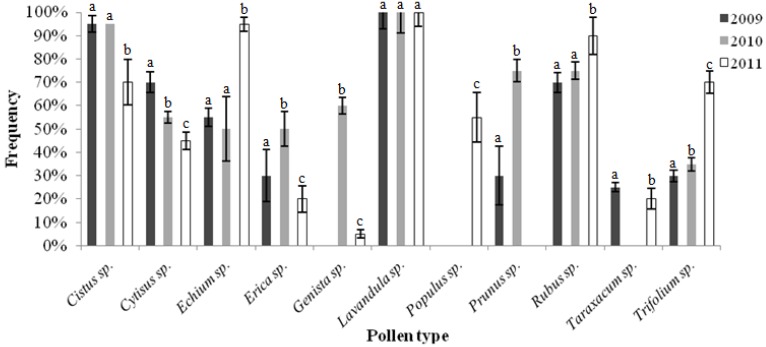
Polinic spectrum of the “Terra Quente” honeys from the three harvests. The letters (a,b,c) represents which honeys are different by Tukey test with significance of α = 0.05.

The analysis of the pollen profile of honey allows inferring its floral origin and confirms the identity of the honey source indicated by the beekeepers. The identified pollen and its frequency on the analyzed honeys from the different harvests are presented in Figure 1. 

According to the “book of specifications”, in the spectrum of the “Terra Quente” honey pollen of the *Papilionaceae* family prevail (*Cytisus multiflorus*, *Cytisus striatus*, *Retama sphaerocarpa*, *Genista florida*), which is due to the wide spread of these botanic species in the Northeast of Portugal [20]. However, the special characteristics of “Terra Quente” honeys are associated with the presence of rosemary (*Lavandula pedunculata*). Our results showed that 55% of the samples harvested in 2009 had more than 35% of *Lavandula pedunculata*, being thereby denominated “rosemary honey”, in agreement with the book of specifications. In the same harvest, 45% of the samples had percentages of *Lavandula* sp. between 15% and 35%. The honey collected in 2010, 5% of the samples had less than 15% of *Lavandula* sp., 30% had between 15% and 35% and 65% had more than 35% of this botanic specie. In the third harvest (2011), 25% of the samples had less than 15% of *Lavandula* sp., half of the samples contained between 15 and 35% and 25% showed more than 35% of this pollen. In the harvest of 2010, the pollen of *Genista* sp. was also very abundant, in contrast to the verified in the other years under study.

### 2.2. Colour Analysis

**Figure 2:**
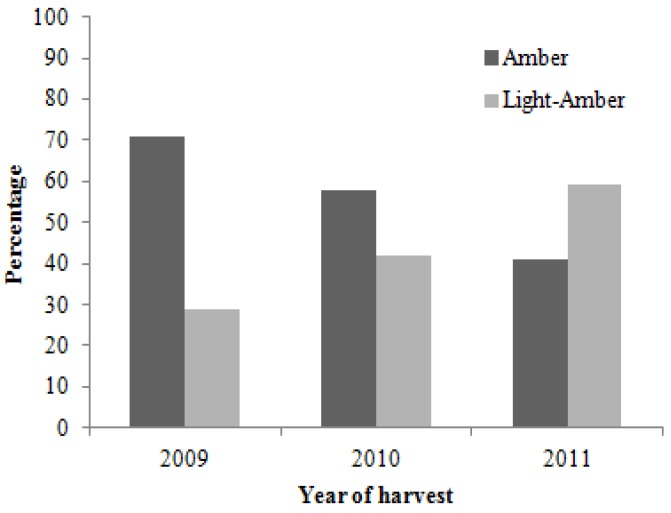
Colour of the samples in each harvest.

Colour is considered as a quality parameter for honey. It depends on the floral origin and the minerals’ content [21]. Light-coloured honeys usually have low ash contents, while dark-colored honeys generally have higher [22]. The colour of the analyzed honey samples varied between light amber to amber. In the honey harvested in 2009 amber honey prevailed (71%), while in 2010 the most abundant was the amber type (41%). 58% of the honey harvested in 2011 was light amber (Figure 2). 

### 2.3. Physicochemical Analyses

The results obtained from the physicochemical parameters are presented in the Table 1. The percentage of moisture ranged between 14.50 and 18.11, with mean values of 15.94 ± 0.70, 16.28 ± 0.46, 16.37 ± 0.70, in samples from 2009, 2010 and 2011, respectively. These results are bellow the limits of <20%, suggesting that the extraction period and the maturation of the product were adequate. In fact, honey moisture content depends on the environmental conditions and the manipulation from beekeepers at the harvest period, and can vary from year to year [23]. High moisture content in honey can increase its water activity and consequently make the preservation and storage more difficult. In addition, it may cause flavor loss and diminution of the “shelf-life”. Concerning this parameter, there were no significant differences, using the Tukey test (*p* < 0.05), that is one of several tests that can be used to determine which means amongst a set of means differ from the rest, between humidity values obtained for the honey samples of the three harvests.

**Table 1:** Physicochemical results of “Terra Quente” honeys from the three harvests.

Harvest (Year)		Parameters								
	Moisture (%)		Electrical Conductivity(mS·cm^−1^)	Ash (%)	HMF(mg·kg^−1^)	Diastase activity(Gothe Scale)	pH	Free acidity(meq·kg^−1^)	Reducing sugars (%)	Apparent sucrose (%)
First (2009)	15.94 ± 0.70 a(14.50–17.21)		0.25 ± 0.07 b(0.13–0.36)	0.12 ± 0.06 b(0.02–0.21)	2.41 ± 1.45 b(1.31–7.02)	14.04 ± 3.51 b(10.06–25.00)	3.58 ± 0.14 a(3.32–3.81)	25.58 ± 6.12 a(13.94–33.63)	69.75 ± 4.19 a(63.71–76.89)	4.59 ± 1.51 a(2.60–7.53)
Second (2010)	16.28 ± 0.46 a(15.40–17.02)		0.23 ± 0.06 b(0.15–0.36)	0.10 ± 0.05 b(0.03–0.21)	4.39 ± 2.64 a(2.63–13.84)	15.09 ± 1.29 ab(13.59–19.17)	3.25 ± 0.26 b(2.93–4.12)	25.16 ± 5.69 a(15.12–38.22)	66.96 ± 2.91 b(62.52–74.11)	4.73 ± 1.61 a(2.44–8.12)
Third (2011)	16.37 ± 0.70 a(15.41–18.11)		0.29 ± 0.04 a(0.26–0.36)	0.15 ± 0.03 a(0.12–0.21)	3.61 ± 1.24 ab(1.71–7.28)	16.95 ± 2.26 a(11.99–19.10)	3.28 ± 0.25 b(2.97–3.64)	28.28 ± 3.93 a(21.52–36.80)	65.59 ± 2.08 b(60.21–68.57)	4.54 ± 1.28 a(2.34–7.36)
*p*-value	0.082		0.006	<0.001	0.006	0.002	<0.001	0.143	<0.001	0.914

Results are given as the mean ± standard deviation (min–max values). The letters (a,b) represents which honeys are different by Tukey test with significance of α = 0.05.

The electrical conductivity of the samples ranged between 0.13 and 0.36 mS·cm^−1^, suggesting that all samples are from nectar honey. This parameter of honey is closely related to the concentration of mineral salts, organic acids and proteins and shows great variability according to the floral origin. It is important for the differentiation of honeys, being therefore used in routine honey quality control [12]. 

The ash content is a complex function of the floral, geographical, soil and climatic characteristics and conditions [23]. The results found (0.02 to 0.21%) are much lower than the reported, by our team [15,16] and others worldwide [14], for other types of honey. This testifies the clearness of DPO honey samples and the lack of adulteration with molasses. The results obtained for electrical conductivity and ashes in the third harvest (2011) differed significantly from the results of the two previous extractions (*p* = 0.006 and *p* = 0.000, for 2010 and 2009).

HMF content and diastase activity are widely recognized as indicators of honeys’ freshness [5]. The evaluation of the two parameters is very important as the changing behavior of diastase activity makes it an uncertain parameter to determine if honey has been submitted to heating [24]. Several factors influence the levels of HMF, such as temperature and time of heating, storage conditions, pH and floral source [25]. It is well known that honey heating results in the formation of HMF, which is produced during acid-catalysed dehydration of hexoses, such as fructose and glucose and Maillard reactions [23]. In fact, our results show that the analyzed honeys are fresh products and ensured that they weren’t submitted to heating or inadequate storing conditions, since the HMF content varied between 1.31 mg·kg^−1^ and 13.84 mg·kg^−1^. Concerning this parameter the highest value was obtained in the harvest of 2010 (4.39 ± 2.46 mg·kg^−1^). In the honey of 2011, the amount of HMF didn’t differ significantly from the obtained in the two previous harvests (*p* = 0.102). However, the values obtained for the first and second harvests were significantly different (*p* = 0.006).

Diastase is an enzyme naturally occurring in honey. Its level depends upon geographic and floral origins of the product. The diastase activity found in the analyzed honeys (2009: 14.04 ± 3.51 mg·kg^−1^; 2010: 15.09 ± 1.29 mg·kg^−1^; 2011: 16.95 ± 2.26 mg·kg^−1^) was higher than 8 in the Gothe Scale (minimal value allowed by the regulations). The second harvest didn’t differ significantly from the others (*p* = 0.065), but there were significant differences between the one from 2009 and the one from 2011 (*p* = 0.002). 

Honey pH is affected by the conditions during extraction and storage, which also influences texture, stability and shelf-life. pH is indeed a useful index to predict the microorganisms’ growth, as the low pH of honey inhibits their presence [26]. It should be noted that honey’s pH is not directly related to the free acidity, due to the buffer action of the acids and minerals, which are naturally present. The results obtained for this parameter ranged from 2.93 and 4.12. These values are consistent with the published reported values of European monofloral honeys [27].

The free acidity didn’t differ significantly among the samples from the different harvests. In the honeys from the first harvest, the mean value was of 25.58 ± 6.12 meq·kg^−1^, in the second it was 25.16 ± 5.69 meq·kg^−^^1^ and in the third 28.28 ± 3.93 meq·kg^−1^. The presence of organic acids in equilibrium with their corresponding lactones, or internal esters, and some inorganic ions, such as phosphate, may influence the free acidity of honey. High acidity can be indicative of fermentation of sugars into organic acids. All the investigated samples met the requirements set by the regulation [1,2], which establish that the acidity shouldn’t exceed 50 meq·kg^−1^.

Concerning the reducing sugars (fructose and glucose), the European Council Directive [1] imposes a limit of reducing sugars ≥60 g/100 g. The percentage of reducing sugars oscillated between 60.21% and 76.89%. The results obtained for this physicochemical parameter do not only meet the standards but also are identical to the observed by other authors in different types of honey [15,26,28].

Apparent sucrose (non-reducing sugar) varied between 2.34% and 8.12%. Significant differences among the samples from different harvests were not noted. This attests that the extraction of the product was adequate and that honey wasn’t adulterated [29].

As mentioned before, all the results were within the limits allowed by the European Council Directive [1] and the Codex Standard for Honey [2]. However, 16 samples (five from the first harvest; five from the second and six from the third) didn’t meet the requirements set by the PDO book of specifications, since in some of them the percentage of moisture was higher than 17% and in others the apparent sucrose exceeded 6%. In addition, some of those samples had less than 65% of reducing sugars. 

Our results suggest that some modifications in the PDO book of specifications should be introduced, in order to avoid devaluation of products that, despite being in agreement with the legislation, don’t exactly fit in the current specifications. This is particularly important as most of the “Terra Quente” PDO honey is exported. However, it is necessary to conduct further studies, since the number of samples tested does not allow generalization.

### 2.4. Microbiological Analyses

Despite the stress conditions found in honey, many microorganisms can grow in this matrix. Microorganisms that survive in honey are those that withstand the concentrated sugar, acidity and other antimicrobial characters of honey. The presence of fungi in honey is linked to contact with the intestinal contents of bees, bee hive and grass. In any case this natural reservoir for microbes does not diminish the many important uses that honey is known for. The results obtained for the parameters that indicate the commercial quality (aerobic mesophiles, moulds and yeasts), sanitary quality (fecal coliforms) and safety (sulphite-reducing clostridia and *Salmonella*) are presented in Table 2.

**Table 2:** Microbial analyses of “Terra Quente” honey samples.

Harvest	Aerobic mesophiles (cfu·g^−^^1^)	Moulds and yeast (cfu·g^−^^1^)	Fecal coliforms (MPN)	Sulphite-reducing clostridia (in 0.01 g)	*Salmonella* (in 25 g)
First	38.20 ± 21.62 a	24.05 ± 11.81 a	<1	Negative	Negative
Second	37.45 ± 19.99 a	30.95 ± 22.16 a	<1	Negative	Negative
Third	37.45 ± 18.27 a	27.45 ± 18.33 a	<1	Negative	Negative
*p*-value	0.681	0.482	n.a.	n.a.	n.a.

Results are given as the mean ± standard deviation. The letter (a) represents that the honeys are not different by Tukey test with significance of α = 0.05.

Aerobic mesophiles, moulds and yeasts in the analyzed honey ranged from 37.45 ± 18.27 to 38.20 ± 21.62 cfu·g^−1^ and from 24.05 ± 11.81 to 30.95 ± 22.16 cfu·g^−1^ for aerobic mesophiles and moulds and yeasts, respectively. No statistical differences were found, for all the analyzed parameters among the different harvests (aerobic mesophiles: *p* = 0.680; moulds and yeasts: *p* = 0.482). These results are generally superior than those reported previously [15] when analyzing commercial Portuguese honeys, what may be due to the pasteurization they go through. However, were lower than the reported by [30] in honeys from Argentina and by [31] in artisanal honeys from the Northeast of Portugal. From the microbiological point of view, these low values of aerobic mesophiles, molds and yeasts are most probably related to the environmental conditions, and are indicative of an appropriate management of apiaries.

All the samples were negative in respect to fecal coliforms, sulphite-reducing clostridia and Salmonella. On the other hand, previous surveys about non-PDO honey reported moderate to high levels of contamination, by coliforms and sulphite-reducing clostridia [30,14]. It should be noticed that the European Union doesn’t have specifications/legislation concerning the microbiological parameters of honey, despite the negative effects that the presence of microorganisms, mainly *C. botulinum* can have in public health, especially when the honey is consumed by children, the elderly and immunodepressed patients. Thus, it is very important to conduct studies in order to establish microbiological criteria and analyses’ methodologies for honey.

### 2.5. Multivariate Analysis

**Figure 3:**
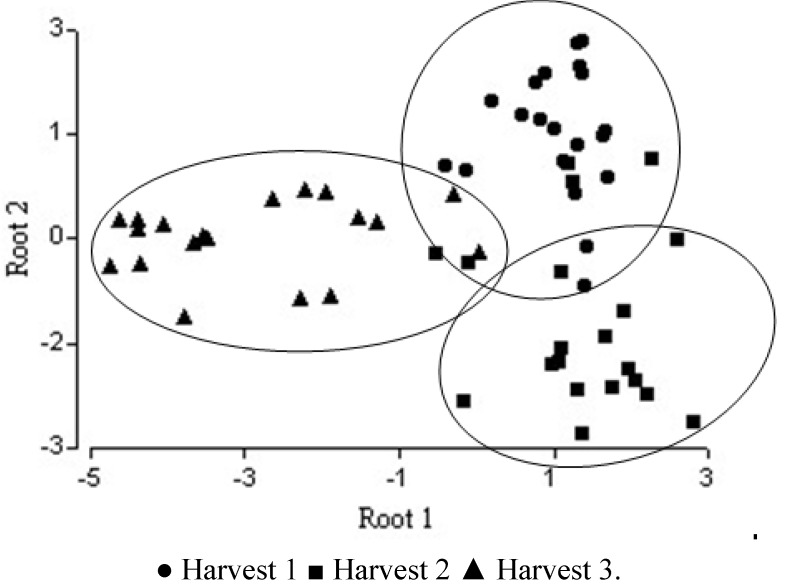
Separation of harvest honeys using palynological analysis: plot of discriminant scores.

After applying the algorithm for selecting variables, the variable “pollen type” showed perfect discriminatory power in stepwise discriminant analysis performed (Table 3) (Wilks’ Lambda = 0.000). The first canonical discriminant function explained 74% of the total variance while the second accounted for 26%. The size of the coefficients indicates the discriminant power of the predictor variables. Thus it can be seen that in the function 1 the variables *Echium* sp., *Cistus* sp. and *Rubus* sp.discriminate best among the three groups. Function 2 was capable of discriminating *Genista* sp. and *Prunus* sp. The plot of the two canonical variables shows a complete separation among the third harvest with respect to the other two. Some samples of the first and second harvest are superposed (Figure 3). 

**Table 3:** Results of stepwise discriminant analysis (SDA) of palynological parameters in the three harvests of PDO honey.

	Wilks’ Lambda	Partial Lambda	F-remove(2,45)	*p*-level	Tolerance	1-Tolerance(R^2^)
*Echium* sp.	0.000	0.847	4.054	0.240	0.561	0.439
*Genista* sp.	0.000	0.693	9.962	0.002	0.836	0.164
*Populus* sp.	0.000	0.714	9.008	0.005	0.158	0.842
*Prunus* sp.	0.000	0.905	2.360	0.106	0.837	0.163
*Rubus* sp.	0.000	0.962	0.898	0.415	0.476	0.524

F-remove = R to remove value.

The physicochemical variables selected by stepwise discriminant analysis as the more discriminant were, in this order, diastase activity, pH, reducing sugars, free acidity and HMF. Wilks’ Lambda, indicates the contribution of each variable to the discrimination, as it can be seen that the five do not surpass 0.5 (Table 4). In this work the significance is high for HMF, free acidity and reducing sugars (*p* < 0.01). From this it can be concluded that the selected parameters have low discriminate power. The behaviour of the standardized canonical coefficients of the found discriminating function, as well as the analysis of the structure of the matrix, showed that the variables with greater weight, in sequence descendent, in the differences between the groups were: pH (0.53), reducing sugars (0.48), and diastase activity (−0.47), free acidity show lower values (0.03). 

**Table 4:** Results of stepwise discriminant analysis (SDA) of physicochemical parameters in the three harvests of PDO honey.

	Wilks’ Lambda	Partial Lambda	F-remove(2,53)	*p*-level	Tolerance	1-Tolerance(R^2^)
HMF	0.485	0.633	15.349	<0.001	0.472	0.528
Diastase activity	0.332	0.924	2.188	0.122	0.795	0.205
pH	0.338	0.908	2.673	0.0783	0.924	0.076
Free acidity	0.383	0.802	6.562	0.003	0.861	0.139
Reducing sugars	0.373	0.823	5.695	0.006	0.466	0.534

F-remove = R to remove value.

**Figure 4:**
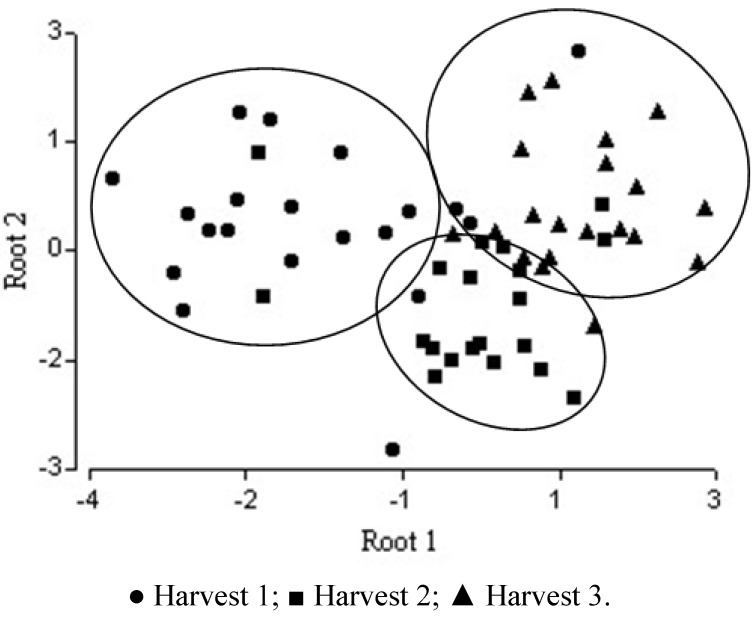
Separation of harvest honeys using physicochemical analysis: plot of discriminant scores.

The general shape of the distribution of tree harvests scores on a scatter diagram whose axes are the first two canonical variables is shown in Figure 4, where the separation is correct for third harvest whereas some samples of first and second are overlapped. The percentage of accumulated variance for the first two functions of the discriminant analysis according to the physicochemical variables is shown in Figure 3.

## 3. Experimental

### 3.1. Chemicals and Materials

All the chemical reagents used were purchased from Sigma Chemical Co. (St. Louis, MO, USA) and were of analytical grade. The culture mediums were purchased from Himedia (Mumbai, India). The water was purified using a Milli-Q purification system (Millipore, Bedford, MA, USA). The spectrophotometer used was the model Unicam UV-Visible Spectrometry Hekios (Thermo Fisher Scientific, Hampshire, UK). An Abbe refractometer (Atago Digital Refractometer, Tokyo, Japan) was also used.

### 3.2. Honey Samples

**Figure 5:**
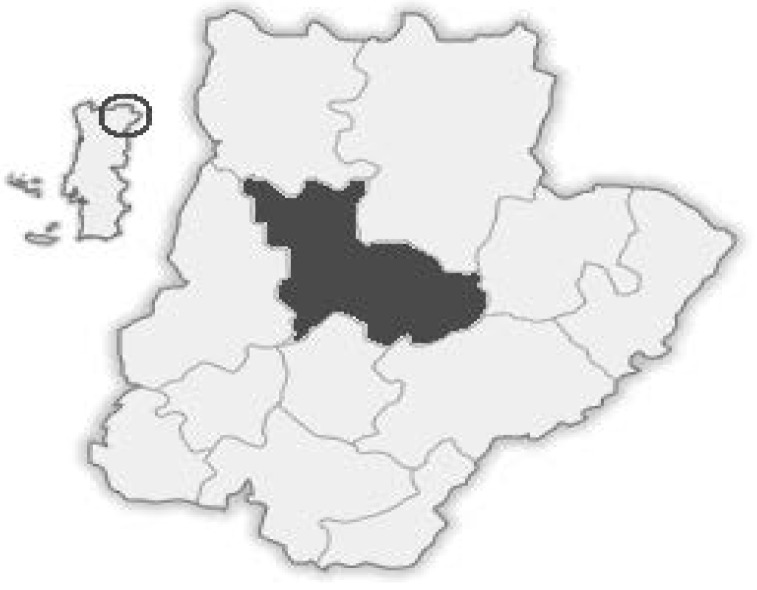
“Terra Quente” region, in which the DOP honey samples were collected.

Sixty honey samples, with Protected Denominations of Origin, were harvested (20 in 2009, 20 in 2010 and 20 in 2011) from different apiaries located in the “Terra Quente” region (Figure 5), that includes many councils from the Northeast of Portugal: Alfândega da Fé, Carrazeda de Ansiães, Macedo de Cavaleiros, Mirandela, Vila Flor and Valpaços. 

The Protected Denomination of Origin of the “Terra Quente” honey was registered in 1994 and the geographical area of production includes 10 councils from the Northeast of Portugal. After honeys’ harvest, the samples were delivered to the Microbiology Lab, where they were stored in a dark place at room temperature (±20 °C) until analysis, which occurred in no more than one month after the extraction from the hives by beekeepers. All the samples showed no sign of fermentation or spoilage.

### 3.3. Pollen Analyses

The samples were subjected to qualitative pollen analysis reported previously in detail [32]. Briefly, pollen grains were extracted from 10 g of crude honey. The sample was then dissolved in distilled water and the sediment was concentrated by repeated centrifuging 30 min at 1,500 rpm. About 10 mL of acetolysis mixture was added and the tubes were incubated in a water bath (100 °C for 3 min), stirred vigorously, centrifuged and decanted. 20 mL of water-free acetic acid was added, stirred, centrifuged, and decanted. The precipitate was washed in distilled water, centrifuged, and decanted. About 12 mL of 7% KOH was added, stirred thoroughly, centrifuged, and decanted. Finally, the pollen grains were stained with a solution of basic fuchsin and mixed with glycerin. The examination of the pollen slides was carried out with an optical microscope at 400× and 1,000× in order to identify the pollen types. 

The recognition of the pollen types was based on the reference collection of the Escola Superior Agrária-Instituto Politecnico de Braganca and different pollen morphology guides. The following terms were used for pollen frequency classes: predominant pollen (P, more than 45% of pollen grains counted), secondary pollen (S, 16–45%) and important minor pollen (I, 3–15%). 

### 3.4. Colour Analyses

Honey samples were heated to 50 °C, in order to dissolve its sugar’s crystals, and the colour was determined spectrometrically (measure of the absorbance of 50% honey solution (w/v) at 635 nm). The colour was determined according to the Pfund scale after conversion of the absorbance values [33]:


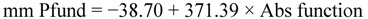
(1)


### 3.5. Physicochemical Analyses

The physicochemical analyses were performed in accordance with the Official Methods of Analysis of Association of Official Analytical Chemists [34] and The Harmonized Methods of the European Honey Commission [35]. Three replicate analyses were made from each sample to obtain the reported data.

#### 3.5.1. Moisture Content

The determination of the moisture content was carried out by refractometry, using an Abbe refractometer. All measurements were performed at 20 °C, after waiting for 6 min for equilibrium, and obtaining the corresponding percentage of moisture (g/100 g honey) from the refractive index of the honey sample by consulting the standard table.

#### 3.5.2. Electrical Conductivity

The determination of the electrical conductivity was performed by the conductimetric assay (WTW Inolab conductivimeter), from a solution containing 10 g of honey in 75 mL of distilled water and reported as such in this publication.

#### 3.5.3. Ash Content

Total ash was estimated by conductimetry using the following equation [36]:


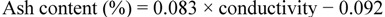
(2)

#### 3.5.4. pH

pH was measured with a combined pH glass electrode connected to pH-meter, in a solution prepared with 10 g of honey in 75 mL of distilled water.

#### 3.5.5. Free Acidity

Free acidity was determined by potentiometric titration. Before further analysis, the honey samples were homogenized in a water bath and filtered through gauze. Ten grams of honey were then dissolved in 75 mL of distilled water, and alcoholic solution of phenolphthalein added. The solution was titrated with 0.1 N NaOH. The milliequivalents of acid per kg of honey were determined as 10 times the volume of NaOH used in titration.

#### 3.5.6. Reducing Sugars and Apparent Sucrose

These parameters were determined by potentiometric titration using the Fehling’s test (Lane and Eyon modified method).

#### 3.5.7. Hydroxymethylfurfural (HMF)

Five grams of honey were dissolved in 25 mL of distilled water, treated with a clarifying agent (0.5 mL of Carrez I and 0.5 mL of Carrez II solutions). The solution was filtered, and the first 10 mL discarded. The absorbance of the filtered solution was measured at 284 and 336 nm against a filtered solution treated with NaHSO_3_. HMF was determined following the equation:


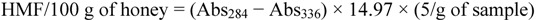
(3)

#### 3.5.8. Diastase Activity

Diastase activity was determined using a buffered solution of soluble starch and honey incubated in a thermostatic bath at 40 °C. Thereafter, 1 mL aliquot of this mixture was removed periodically and the absorption of the sample was followed at 660 nm. The diastase value was calculated using the time taken for the absorbance to reach 0.235, and the results were expressed in Gothe degrees as the amount (mL) of 1% starch hydrolyzed by the enzyme diastase in 1 g of honey in 1 h. 

### 3.6. Microbiological Determinations

The microbiological analyses of the samples were carried out as described previously [15]. In order to evaluate the microbial quality of this natural product it were researched the commercial quality parameters: mesophilic microorganisms and yeasts and moulds, indicators of sanitary quality: fecal coliforms and *E. coli*, indicators of safety: sulphite reducing clostridium spores and *Salmonella*. All microbial tests were performed in triplicate.

#### 3.6.1. Sample Preparation

Ten g of honey were aseptically taken and homogenized for 3 min with 90 mL of pre-chilled (4 ± 0.5 °C) sterile peptone-physiological saline solution (0.1% neutral peptone + 0.85% NaCl in sterile deionized H_2_O, pH = 7.0 ± 0.05). Decimal serial dilutions were prepared from this homogenate in the same chilled sterile diluents (1:10, by vol).

#### 3.6.2. Enumeration of the Total Mesophilic Microorganisms

Aerobic mesophilic bacteria were counted by incorporation of 1 mL of each dilution into standard Plate Count Agar (PCA) and incubated at 30 °C for 48 h. Microbial counts were expressed as colony-forming units per gram of honey (cfu·g^−^^1^).

#### 3.6.3. Enumeration of Yeast and Moulds

Moulds and yeasts enumeration was made on DG18 and incubated at 25 °C for 5 days. Microbial counts were expressed as cfu·g^−^^1^.

#### 3.6.4. Sulphite Reducing *Clostridium* Spores

For sulphite-reducing clostridia counting, aliquots of 10, 5, 1 and 0.1 mL of the initial suspension were added to an empty tube, thermally treated at 80 °C for 15 min and covered with Differential Reinforced Clostridial Broth (DRCM), and incubated at 37 °C for 5 days. At the end, the black colonies were counted. The results are expressed as presence of sulphite-reducing clostridia in 0.01 g. Results were expressed as most probable numbers of sulphite-reducing *Clostridium* spores per gram of honey (MPN·g^−1^). 

#### 3.6.5. Fecal Coliforms and *Escherichia Coli*

The presence of coliforms, fecal coliforms and *E. coli* in honey may be determined by means of the Most Probable Number (MPN) procedure. Briefly, this method involves serially diluting out the target organisms in the sample, in 5-replicate aliquots, to extinction. The probable level of the target organisms is then statistically estimated from a Hoskins table. Gas production is used as an indication of ability to ferment lactose from Lauryl Tryptose (LST) Broth (presumptive coliform test); gas production from Brilliant Green Lactose Bile (BGLB) broth is considered confirmation of coliform presence; gas production at 45 °C from *E. coli* broth is used as confirmation of fecal coliform presence; and appearance of typical nucleated, dark-centred colonies with or without metallic sheen when positive EC broths are streaked onto Eosin methylene blue (L-EMB) Agar are indicative of *E. coli*. The typical colonies on L-EMB agar must be isolated in PCA and then further biochemical tests are used to prove the presence of *E. coli*. Results were expressed as MPN g^−1^.

#### 3.6.6. *Salmonella*

The detection of *Salmonella* has 4 steps that include pre-enrichment (Bulfered Peptone Water), selective enrichment (Rappaport Vassiliadi and Muller-Kauffmann Tetrathionate-Novobiocin Broth-MKTTn), planting out (Xylose lysine Deoxycholate Agar-XLD agar) and confirmation Nutrient Agar. Afterwards, biochemical confirmations are performed. Results were expressed as absence or presence of *Salmonella* in 25 gram of honey.

#### 3.6.7. *Staphylococcus Aureus*

Serial dilutions of the sample were inoculated in Baird-Parker Broth with Egg Yolk Tellurite and Sulfadimidine Solution during 24 h (37 °C). After, three to five characteristic colonies were selected, in order to verify the presence of coagulase and catalase. Microbial counts were expressed as colony-forming units per gram of honey (cfu·g^−^^1^). 

### 3.7. Statistical Analyses

Results are shown as mean values and standard deviation. Prior to the statistical analysis, normality tests (Shapiro-Wilk, Anderson-Darling, Liliefors and Jarque-Bera) were carried out, considering each harvest independently or the three harvests as a whole. We verified that the results follow a normal distribution. For the statistical treatment of the results an one-way analysis of variance (ANOVA), followed by Tukey’s HSD Test with α = 0.05, was applied together with a discriminant function analysis, to determine which variables (physicochemical parameters and palynological characteristics of honey samples) discriminant between two or more naturally occurring groups (three different harvests). In stepwise discriminant function analysis (Forward Stepwise Analysis), a model of discrimination is built step-by-step. Specifically, at each step all variables are reviewed and evaluated to determine which one will contribute most to the discrimination between groups. That variable will then be included in the model and the process starts again. A canonical correlation analysis was performed determining the two functions and canonical roots. The summary statistics for all variables in the model are: (i) Wilk’s Lambda, that measures deviations within each group with respect to the total deviations without distinction of groups, it assumes values between 1.0 = no discriminatory power; 0.0 = perfect discriminatory power; (ii) partial Lambda is the Wilk’s Lambda associated with the unique contribution of the respective variable to the discriminatory power of the model; (iii) F to remove, which is the F-value associated with the respective partial Wilk’s Lambda; (iv) p-level is the p-level associated with the respective F to remove; (v) tolerance (R^2^) is the R^2^-value of the respective variable with all other variables in the model. The statistical treatments were carried out using the programs SAS v. 9.1.3 (SAS Institute Inc., Cary, NC, USA) and STATISTICA (StatSoft Inc., Tulsa, OK, USA).

## 4. Conclusions

Within the scope of this first study on “Terra Quente” honey with Protected Denomination of Origin, the characterization of 60 samples was carried out based on pollen analyses and some physicochemical and microbiological parameters. Overall it can be concluded that the analyzed honeys, harvested in three consecutive years, were of high quality, since all the values obtained for the physicochemical and microbiological parameters exceed in quality the limits imposed by the present legislation. Indeed, almost all the analyzed samples had high diastase activity and a low HMF content, suggesting that they were fresh products, unaltered and well handled. In addition, the sugar content, whose anomalous values may be reliable indicators of adulteration, was, generally, within the DOP limits. The pollen from *Lavandula pedunculata* was found in all the analyzed samples, consequently it was the pollens (*Populus* sp., *Echium* sp., *Cistus* sp. and *Rubus* sp.) that best discriminated the groups. Multivariate analysis revealed that the most important physicochemical parameters were: diastase activity, pH and reducing sugars. Even though all the samples met the requirements of the legislation, some of them didn’t comply with the PDO specifications. In addition, these specifications do not list parameters that are very important in the assessment of honeys’ quality, such as pH and microbiological status, thus further trials are required in order to supplement and update the PDO book of specifications. This would play a key role in the further valorization of “Terra Quente” honey, particularly in the international market. 
